# The Role of Obesity in Sepsis Outcome among Critically Ill Patients: A Retrospective Cohort Analysis

**DOI:** 10.1155/2016/5941279

**Published:** 2016-09-29

**Authors:** Matthaios Papadimitriou-Olivgeris, Diamanto Aretha, Anastasia Zotou, Kyriaki Koutsileou, Aikaterini Zbouki, Aikaterini Lefkaditi, Christina Sklavou, Markos Marangos, Fotini Fligou

**Affiliations:** ^1^Division of Infectious Diseases, School of Medicine, University of Patras, Rion, 26504 Patras, Greece; ^2^Department of Anaesthesiology and Intensive Care Medicine, School of Medicine, University of Patras, Rion, 26504 Patras, Greece

## Abstract

*Background.* The objective of this study was to assess the correlation between sepsis, obesity, and mortality of patients admitted to an Intensive Care Unit (ICU).* Subjects and Methods.* Data of all patients admitted to the ICU of a tertiary hospital during a 28-month period were retrospectively analyzed and included in the study.* Results.* Of 834 patients included, 163 (19.5%) were obese, while 25 (3.0%) were morbidly obese. Number of comorbidities (*P* < 0.001), bloodstream infection (*P*  0.033), and carbapenemase-producing* Klebsiella pneumoniae* colonization during ICU stay (*P*  0.005) were significantly associated with obesity, while nonobese patients suffered more frequently from spontaneous intracranial hemorrhage (*P*  0.038). Total ICU mortality was 22.5%. Increased mortality among obese ICU patients was observed. Sepsis was the main condition of admission for which obese patients had statistically lower survival than normal weight subjects (76.3% versus 43.7%; *P*  0.001). Mortality of septic patients upon admission was independently associated with SOFA score upon ICU admission (*P*  0.003), obesity (*P*  0.014), pneumonia (*P*  0.038), and development of septic shock (*P*  0.015).* Conclusions.* Our study revealed that sepsis upon ICU admission is adversely influenced by obesity but further studies are needed in order to assess the role of obesity in sepsis outcome.

## 1. Introduction

Obesity is attaining epidemic proportions in Europe especially Greece [1, 2]. During the last decades obesity prevalence has increased significantly, especially among children and adolescents [1, 3]. This increase that began in the 1980s was attenuated over the last eight years [3]. Globally, an increase by 27.5% was observed in prevalence of overweight and obesity combined between 1980 and 2013 [3]. Nowadays, 71% of male and 51% of female adult Greeks are obese or overweight [3]. The prevalence of obesity was 28% and 26% among Greek men and women, respectively [4]. Obese patients are at increased risk of developing comorbidities, such as hypertension, coronary disease, chronic obstructive pulmonary disease, and diabetes [5]. In order to assess the presence of obesity the body mass index (BMI) is used and interpreted according to World Health Organization [6].

The relationship between obesity and mortality of critically ill patients remains unknown, since studies assessing the role of obesity in mortality among patients admitted to intensive care units (ICUs) show contradictory results [7, 8]. Due to comorbidities, several studies reported higher mortality rates among obese critically ill patients. On the contrary, recent studies found lower mortality in obese than in normal weight ICU patients [9, 10], a phenomenon referred to as the “obesity paradox” [11].

This paradox was also observed in subgroups of critically ill patients, such as patients with septic shock or those with peritonitis [12, 13]. The etiology for this paradox is not clear and can be due to selection bias in the study design or differences in patients' characteristics [11].

The aims of this study were to describe the epidemiology of obesity among critically ill patients hospitalized in a Greek ICU, to assess its effect on ICU mortality and to investigate the correlation between sepsis and obesity.

## 2. Subjects and Methods

### 2.1. Patients and Data Collection

This single-center retrospective study was performed in the general ICU of the University General Hospital of Patras (UGHP), Greece. UGHP is a tertiary hospital that accepts patients for the region of Western Greece, Peloponnese, and Ionian Islands and a population reaching one million people, whereas the ICU is separated in two compartments of ten and three beds, respectively. In the main compartment, two isolation and two semi-isolation beds are available. The medical records of all adult patients (≥18 years) that were admitted from November 2011 to February 2014 were reviewed until their discharge from the ICU. The study was approved from the Ethical Committee of the University Hospital of Patras (number 571). The need for informed consent was waived because of the retrospective and observational design of the study according to European legislation.

Patient data (epidemiologic data, comorbidities, colonization/infection, antimicrobial administration, and ICU procedures) were prospectively collected and recorded in the ICU computerized database (Criticus*™*, University of Patras, Greece). Severity scores of illness [APACHE II (Acute Physiology and Chronic Health Evaluation II), SAPS (Simplified Acute Physiology Score II), and SOFA (Sequential Organ Failure Assessment)] were calculated upon admission. Patients were included when they were aged 18 years or older. We excluded pregnant women and cardiac surgery patients. BMI was calculated for most of the patients upon ICU admission while nurses using a nonrigid measuring tape measured their height. According to the WHO criteria patients were categorized in five groups: underweight (<18.5 kg/m^2^), normal weight (18.5–24.9 kg/m^2^), overweight (25–29.9 kg/m^2^), obese (30–39.9 kg/m^2^), and morbidly obese (≥40 kg/m^2^) [6]. Patients were categorized into the following groups according to the main reason for admission: trauma, spontaneous intracranial hemorrhage, sepsis, postoperative observation, respiratory insufficiency, and others (com*α*, epilepsy, intoxication, etc.). Sepsis and septic shock were defined according to the Third International Consensus Definitions for Sepsis and Septic Shock [14].

### 2.2. Statistical Analysis

Statistical analysis was performed with SPSS version 22.0 software package (IBM SPSS Statistics for Mac, Version 22.0, Armonk, NY, USA). For the statistical analyses patients were further categorized in two groups: obese patients (BMI ≥ 30 kg/m^2^) and nonobese patients (BMI < 30 kg/m^2^). Categorical variables were analyzed by using the Fisher exact test or chi^2^ test while continuous variables were analyzed with Mann-Whitney *U* test or one-way ANOVA, as appropriate. Three different analyses were performed resulting from a predefined analysis plan. The first one was aimed at determining factors that differ among obese and nonobese patients. The second one was aimed at detecting predictors of ICU mortality of patients that were septic upon admission and the third one was aimed at determining the factors that differ among obese and nonobese septic patients. Backward stepwise multiple logistic regression analysis used all those variables from the univariate analysis with *P* < 0.05. In order to identify factors that were highly correlated, collinearity diagnostics were performed. No factors contributing to multicollinearity were revealed (tolerance > 0.2 and VIF < 10 for all the factors analyzed). All statistic tests were 2-tailed and *P* < 0.05 was considered statistically significant.

## 3. Results

Of the 834 patients, 163 (19.5%) were obese and among them 25 (3.0%) were morbidly obese.  Table 1 shows the univariate analysis of differences among obese and nonobese patients. Sixteen out of 38 factors were found to be statistically significant by univariate analysis (female gender, number of chronic diseases, diabetes mellitus, chronic obstructive disease, spontaneous intracranial hemorrhage, sepsis, ICU length of stay, ICU mortality, number of antibiotics administered, dialysis, enteral nutrition, KPC-Kp colonization, bloodstream infection and septic shock during ICU stay, KPC-Kp infection, and Candida infection during ICU stay). Multivariate analysis revealed that number of chronic diseases (*P* < 0.001; OR 3.2; 95% CI 2.6–3.9), bloodstream infection during ICU stay (*P*  0.033; OR 2.0; 95% CI 1.1–3.7), and KPC-producing* Klebsiella pneumoniae* colonization during ICU stay (*P*  0.005; OR 2.2; 95% CI 1.3–3.7) were significantly associated with obesity, while nonobese patients suffer more frequently than obese patients from spontaneous intracranial hemorrhage (*P*  0.038; OR 0.22; 95% CI 0.05–0.92).

Total ICU mortality was 22.5% (188 patients). Obese patients were characterized by increased ICU mortality, as compared to nonobese ones (28.8% versus 21.0%; *P*  0.036).  Table 2 shows the distribution of ICU patients according to the main reason for admission, BMI, and survival. Sepsis was the main condition of admission for which obese patients had statistically lower survival than normal weight subjects (76.3% versus 43.7%; *P*  0.001).

**Figure 1:**
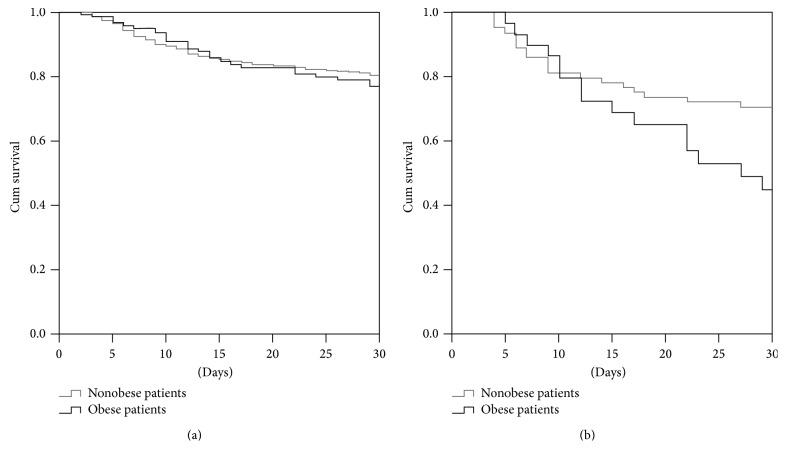
Kaplan-Meier curves of 30-day survival probability according to the presence of obesity of (a) of all patients and (b) septic patients upon ICU admission.

In order to determine the predictors of mortality among septic patients upon ICU admission (125 patients), a second analysis was conducted (Table 3) by comparing survivors (58 patients) and nonsurvivors (67 patients). Univariate analysis revealed 12 statistically significant factors (number of chronic diseases, malignancy, cortisone use, obesity, SAPS II and SOFA score, parenteral nutrition, dialysis, meningitis, pneumonia, urinary-tract infection, and septic shock). Multivariate analysis revealed that SOFA score upon ICU admission (*P*  0.003; OR 1.3; 95% CI 1.1–1.5), obesity (*P*  0.014; OR 5.3; 95% CI 1.4–20.2), pneumonia (*P*  0.038; OR 3.5; 95% CI 1.1–11.3), and development of septic shock (*P*  0.015; OR 3.4; 95% CI 1.3–9.1) were all independently associated with mortality, while urinary-tract infection (*P*  0.010; OR 0.06; 95% CI 0.01–0.52) was associated with survival. Since obesity was an independent predictor of mortality among septic patients upon ICU admission, a further analysis was performed to assess the differences among obese and nonobese patients (Table 4). Twelve out of 34 factors were found to be statistically significant by univariate analysis (ICU length of stay, mortality, cortisone use, number of antibiotics administered, mean antibiotic use per day, tracheostomy, number of invasive catheters, parenteral and enteral nutrition, dialysis, bloodstream infection, and KPC-Kp colonization during ICU stay). In multivariate analysis mortality (*P*  0.002; OR 4.9; 95% CI 1.8–13.2), bloodstream infection upon ICU admission (*P*  0.042; OR 3.1; 95% CI 1.0–9.0), and KPC-producing* K. pneumoniae* colonization during ICU stay (*P*  0.001; OR 2.1; 95% CI 1.3–3.2) were significantly associated with obese septic patients. According to Kaplan-Meier curves the 30-day survival probability is lower in obese septic patients compared to nonobese ones (Figure 1).

## 4. Discussion

The percentages of overweight (39.9%) and obese (19.5%) patients admitted to the ICU were similar to that reported from previous prevalence studies in Greek population [3–5]. Obese patients suffered more often from chronic diseases as compared to nonobese patients, especially diabetes mellitus and chronic obstructive pulmonary disease, while male gender was more common among nonobese patients admitted to our ICU [2, 4]. An interesting finding of our study, reported for the first time, was that spontaneous intracranial (subarachnoid or intraparenchymal) hemorrhage was independently more common in nonobese patients. This contradicts the fact that obesity is a well-known risk factor for nontraumatic brain hemorrhage [15]. The low percentage of obese patients with brain hemorrhage in our study probably does not reflect the reality since, in the present study, only patients admitted to the ICU were included and not patients hospitalized in other hospital wards such as the neurosurgery department. Moreover, this is a retrospective study and further studies are needed in order to elucidate this association. The relatively high percentage of patients with intracranial hemorrhage admitted to the ICU can be explained by the fact that our hospital is the only tertiary hospital, with a neurosurgical department, in a region of one million people. All patients with intracranial hemorrhage diagnosed in other hospitals are transferred to our hospital.

In our study obese critically ill patients had lower ICU survival as compared to nonobese patients, in contrary to the results of previous studies that showed that obesity has a beneficial effect on ICU mortality [9, 10]. This phenomenon, which is known as “obesity paradox”, has no apparent physiological explanation [11]. In a large cohort study Abhyankar et al. found that overweight and obese patients had higher survival rate both thirty days and one year after ICU admission [10]. In our study only obese patients admitted for sepsis presented higher mortality rate compared to nonobese patients, while no difference in outcome was observed in obese patients with other admission reasons. Like previous studies our study also showed that obesity did not influence mortality in patients admitted to the ICU for postoperative observation (nonobese 10.6% versus obese 4.2%, *P*  0.115) [16]. Moreover, the same observation was made for patients admitted after emergency surgery (25.2% versus 34.4%, *P*  0.376) [17].

Obesity has been identified as a risk factor for the development of nosocomial and community-acquired infections [18], but the relation between obesity and infection in critically ill patients is unclear [19]. In our study obesity was associated with infection upon admission and bacteremia during ICU stay. Dossett et al. have also shown that the rate of primary or catheter related bloodstream infection was significantly more common in obese as compared to nonobese patients [20]. Obesity has been seldom identified as a risk factor for colonization by multidrug resistant pathogens [21]. It is noteworthy in the present study that obese patients during ICU stay became more commonly colonized by KPC-producing* K. pneumoniae*, which resulted in higher incidence of infections provoked by the same pathogen. KPC-producing* K. pneumoniae* is prevalent in Greek ICUs causing infections associated with increased mortality [22]. Even though it can be argued that the high colonization rate among obese patients may be due to the prolonged length of stay of these patients (18.4 versus 8.8 days; *P*  0.004), we found that the length of stay until colonization by the aforementioned pathogen did not differ among obese and nonobese patients (9.6 versus 9.0 days; *P*  0.583), indicating that the length of stay plays no role in the colonization incidence. The higher rate of colonization and subsequent infection by KPC-producing* K. pneumoniae* may explain the increased antibiotic administration among critically ill obese patients (Table 1), especially those used for the treatment of aforementioned infection, such as colistin, aminoglycosides, and tigecycline [23]. Obese patients received more commonly antifungal treatment, which can be explained by the increased length of stay of obese patients in the ICU and the higher rate of* Candida* infection. The latter is due to the fact that obesity predisposes to* Candida* species colonization which makes obese patients susceptible to candidemia [23].

Our study shows higher mortality rates in obese septic patients upon ICU admission as compared to nonobese ones. Even though obesity can be expected to be a predictor of mortality among septic patients, this topic remains a subject of considerable debate [12, 24]. A previous retrospective study of patients with septic shock found that obesity was associated with lower mortality, an association that could be influenced by the difference of age and comorbidities among groups [12]. Our study contradicts the results of the aforementioned study. The true effect of obesity on increased mortality, in our study, can be also supported by the fact that obese and nonobese septic patients had the same age and severity of illness upon ICU admission as it is depicted by APACHE II Score, SAPS II, and SOFA score while they also suffered from the same number of comorbidities and had similar rates of septic shock upon ICU admission. In the subgroup of patients admitted with pneumonia (66 patients), mortality was higher among obese patients (56.8% versus 83.3%; *P*  0.078). On the contrary a previous study showed a protective effect of obesity on mortality from community-acquired pneumonia [25]. This discrepancy of results can be explained by the fact that in our cohort study some cases of pneumonia were nosocomial and only severe cases of pneumonia warranting ICU admission were included.

A great percentage of patients (373/834) were admitted to the ICU for postoperative observation. There were similar rates of postoperative admission between obese and nonobese patients while the types of surgeries were the same. The operations included were abdominal, cardiovascular, neurosurgical, and orthopedic and no difference concerning the type of surgery was observed.

Our study has several limitations. Although we included all patients admitted to the ICU during a 28-month period, this is a single-center retrospective study. A second limitation of our study would be the fact that although the study included critically ill patients from a region of one million people, with high rates of multidrug resistant pathogens, our data cannot be extrapolated to patients worldwide. Finally, measurement of height or weight in recumbent patients is associated with errors arising from the presence of numerous attachments or volume depletion or overload.

## 5. Conclusion

In our study prevalence of obesity among critically ill patients reflects prevalence of obesity of Greek population. Obesity was associated with higher incidence of infection and lower incidence of nontraumatic brain hemorrhage while, among patients with sepsis upon ICU admission, obesity was associated with higher mortality. The obesity paradox (lower mortality) was not observed in Greek critically ill patients probably due to the fact that obese ICU patients develop more commonly infections, especially by KPC-producing* K. pneumoniae*, which are associated with reduced survival. More studies are needed in order to evaluate the relationship between obesity and colonization or infection by multidrug resistant pathogens.

## Figures and Tables

**Table 1:** Univariate and multivariate analysis of factors that differ among obese and nonobese critically ill patients. *P* depicts the univariate analysis results while (*∗*) denotes factors that differ in the multivariate analysis too (*P* < 0.001 for number of chronic diseases, *P* = 0.033 for bloodstream infection during ICU stay, *P* 0.005 for KPC-producing *Klebsiella pneumoniae* colonization during ICU stay, and *P* 0.038 for spontaneous intracranial hemorrhage).

Patient characteristics	Nonobese patients (*n* = 671)	Obese patients (*n* = 163)	*P*
*Demographics*			
Age (years)	56.3 ± 19.8	59.1 ± 14.8	0.095
Female gender	129 (32.2%)	44 (43.6%)	0.035
*Chronic diseases (number)*	0.7 ± 0.9	1.0 ± 1.1	0.001^*∗*^
Diabetes mellitus	82 (12.2%)	56 (34.4%)	<0.001
Chronic obstructive pulmonary disease	63 (9.4%)	39 (23.9%)	<0.001
Chronic heart failure	71 (10.6%)	20 (12.3%)	0.575
Chronic renal failure requiring dialysis	38 (5.7%)	11 (6.7%)	0.580
Malignancy (solid organ or heamatologic one)	162 (24.1%)	33 (20.2%)	0.305
Cortisone use (within last month of ICU admission)	56 (8.3%)	9 (5.5%)	0.258
*Reasons for admission*			
Spontaneous intracranial hemorrhage	85 (12.7%)	2 (1.2%)	<0.001^*∗*^
Sepsis	87 (13.0%)	38 (23.3%)	0.001
Respiratory insufficiency	63 (9.4%)	19 (11.7%)	0.463
Postoperative observation	302 (45.0%)	71 (43.6%)	0.792
Trauma	103 (15.4%)	22 (13.5%)	0.625
Others^a^	31 (4.6%)	11 (6.7%)	0.316
*Hospitalization data*			
Prior emergency surgery	218 (32.5%)	49 (30.1%)	0.576
Prior abdominal surgery	194 (28.9%)	49 (30.1%)	0.774
Prior hospitalization	237 (35.3%)	57 (35.0%)	1.000
APACHE II Score upon admission	13.3 ± 7.5	12.8 ± 7.1	0.679
SAPS II upon admission	32.6 ± 13.8	33.8 ± 13.1	0.412
SOFA score upon admission	6.4 ± 3.8	7.0 ± 3.5	0.051
ICU length of stay (days)	8.8 ± 11.4	18.4 ± 9.7	0.004
ICU mortality	141 (21.0%)	47 (28.8%)	0.036
*ICU data*			
Cortisone	225 (33.5%)	64 (29.3%)	0.170
** **Antibiotics administered (number)	2.4 ± 1.6	2.9 ± 2.2	0.036
Mean antibiotic use per day	1.9 ± 1.0	2.1 ± 1.2	0.088
Tracheostomy	183 (27.3%)	55 (33.7%)	0.122
Dialysis	27 (4.0%)	16 (9.8%)	0.005
Parenteral nutrition	110 (16.4%)	38 (23.3%)	0.051
Enteral nutrition	183 (27.3%)	59 (36.2%)	0.027
Number of invasive catheters^b^	0.8 ± 0.9	1.0 ± 1.4	0.253
*Colonization/infection data*			
KPC-Kp colonization during ICU stay	134 (20.0%)	52 (31.9%)	0.002^*∗*^
Days until colonization	9.0 ± 3.1	9.6 ± 3.6	0.583
VRE colonization during ICU stay	24 (3.6%)	8 (4.9%)	0.493
Bloodstream infection during ICU stay	61 (9.1%)	35 (21.5%)	<0.001^*∗*^
Septic shock during ICU stay	91 (13.6%)	45 (27.6%)	<0.001
KPC-Kp infection during ICU stay	29 (4.3%)	19 (11.7%)	0.001
Candida infection during ICU stay	9 (1.3%)	7 (4.3%)	0.022

Data are number (%) of patients or mean ± SD.

ICU: intensive care unit; APACHE II: Acute Physiology and Chronic Health Evaluation II; SAPS: Simplified Acute Physiology Score II; SOFA: Sequential Organ Failure Assessment; KPC-Kp: KPC-producing *K. pneumoniae*; VRE: vancomycin-resistant *Enterococcus*.

^a^Coma, epilepsy, myocardial infarction, and intoxication.

^b^All patients after ICU admission were intubated and mechanically ventilated and were continuously monitored with a central venous catheter, an arterial catheter, and a urinary catheter. Number of catheters does not include the aforementioned ones.

**Table 2:** Distribution of intensive care unit patients according to the main reason for admission, BMI, and survival. *P* denotes the difference in survival between obese and nonobese patients.

Reasons for admission	Nonobese (BMI ≤ 29.9)				Obese (BMI ≥ 30)			*P*
	BMI ≤ 18.5	18.6 ≤ BMI ≥ 24.9	25 ≤ BMI ≥ 29.9	All	30 ≤ BMI ≥ 39.9	BMI ≥ 40	All	
Spontaneous intracranial hemorrhage (87)	0 (0%)	32 (25%)	53 (28%)	85 (27%)	2 (50%)	0 (0%)	2 (50%)	1.000
Sepsis (125)	0 (0%)	40 (35%)	47 (51%)	87 (44%)	33 (85%)	5 (20%)	38 (76%)	<0.001
Respiratory insufficiency (82)	4 (50%)	25 (20%)	34 (41%)	63 (33%)	14 (50%)	5 (0%)	19 (37%)	0.788
Postoperative observation (373)	5 (0%)	159 (9%)	138 (13%)	302 (11%)	60 (2%)	11 (18%)	71 (4%)	0.115
Trauma (125)	0 (0%)	63 (17%)	40 (15%)	103 (17%)	20 (0%)	2 (100%)	22 (9%)	0.734
Others^a^ (42)	0 (0%)	10 (40%)	21 (29%)	31 (32%)	9 (33%)	2 (100%)	11 (45%)	0.481
All (834)	9 (22%)	329 (17%)	333 (25%)	671 (21%)	138 (29%)	25 (28%)	163 (29%)	0.037

Data are number or patients that survived/deceased.

BMI (kg/m^2^).

^a^Coma, epilepsy, myocardial infarction, and intoxication.

**Table 3:** Univariate and multivariate analysis ofpredictors of mortality among septic patients upon intensive care unit admission. *P* depicts the univariate analysis results while (*∗*) denotes factors that differ in the multivariate analysis too (*P* 0.003 for SOFA score upon admission, *P* 0.014 for obesity, *P* 0.038 for pneumonia, and *P* 0.015 for septic shock).

Patient characteristics	Survivors (*n* = 58)	Nonsurvivors (*n* = 67)	*P*
*Demographics*			
Age (years)	59.0 ± 19.7	61.0 ± 17.1	0.603
Male gender	28 (57.1%)	27 (57.4%)	1.000
*Chronic diseases (number)*	0.9 ± 1.0	1.6 ± 1.2	<0.001
Diabetes mellitus	10 (17.2%)	17 (25.4%)	0.286
Chronic obstructive pulmonary disease	12 (20.7%)	16 (23.9%)	0.830
Chronic heart failure	8 (13.8%)	9 (13.4%)	1.000
Chronic renal failure requiring dialysis	5 (8.6%)	4 (6.0%)	0.732
Malignancy (solid organ or heamatologic one)	2 (3.4%)	13 (19.4%)	0.006
Cortisone use (within last month of ICU admission)	3 (5.2%)	13 (19.4%)	0.029
Obesity	9 (15.5%)	29 (43.3%)	0.001^*∗*^
*Hospitalization data*			
Prior emergency surgery	14 (24.1%)	15 (22.4%)	0.835
Prior abdominal surgery	17 (29.3%)	17 (25.4%)	0.689
Prior hospitalization	36 (62.1%)	49 (73.1%)	0.249
APACHE II Score upon admission	16.9 ± 7.2	19.1 ± 8.4	0.323
SAPS II upon admission	39.8 ± 12.9	46.3 ± 12.8	0.007
SOFA score upon admission	8.1 ± 3.1	9.9 ± 4.2	0.037^*∗*^
ICU length of stay (days)	14.5 ± 7.2	17.0 ± 17.4	0.216
*ICU data*			
Cortisone	32 (55.2%)	48 (71.6%)	0.064
Antibiotics administered (number)	3.5 ± 1.8	3.9 ± 1.9	0.149
Mean antibiotic use per day	2.8 ± 0.9	2.9 ± 0.9	0.539
Tracheostomy	25 (43.1%)	32 (47.8%)	0.719
Number of invasive catheters^a^	0.8 ± 1.4	1.1 ± 1.5	0.072
Parenteral nutrition	11 (19.0%)	24 (35.8%)	0.046
Enteral nutrition	27 (46.6%)	35 (52.2%)	0.592
Dialysis	3 (5.2%)	13 (19.4%)	0.029
*Infection upon admission data*			
Site of infection			
Meningitis	8 (13.8%)	2 (3.0%)	0.044
Pneumonia	22 (37.9%)	40 (59.7%)	0.020^*∗*^
Intra-abdominal infection	13 (22.4%)	14 (20.9%)	1.000
Urinary-tract infection	12 (20.7%)	3 (4.5%)	0.011
Skin and soft tissue infection	3 (5.2%)	7 (10.4%)	0.337
Bloodstream infection	6 (10.3%)	16 (23.9%)	0.060
Septic shock	8 (13.8%)	47 (70.1%)	<0.001^*∗*^
*Colonization data*			
KPC-Kp colonization during ICU stay	20 (34.5%)	21 (35.8%)	1.000
VRE colonization during ICU stay	5 (8.6%)	6 (9.0%)	1.000

Data are number (%) of patients or mean ± SD.

ICU: intensive care unit; APACHE II: Acute Physiology and Chronic Health Evaluation II; SAPS: Simplified Acute Physiology Score II; SOFA: Sequential Organ Failure Assessment; KPC-Kp: KPC-producing *K. pneumoniae*; VRE: vancomycin-resistant *Enterococcus*.

^a^All patients after ICU admission were intubated and mechanically ventilated and were continuously monitored with a central venous catheter, an arterial catheter, and a urinary catheter. Number of catheters does not include the aforementioned catheters.

**Table 4:** Univariate and multivariate analysis fordifference among obese and nonobese septic patients upon intensive care unit admission. *P* depicts the univariate analysis results while (*∗*) denotes factors that differ in the multivariate analysis too (*P* 0.002 for mortality, *P* 0.042 for bloodstream infection upon ICU admission, and *P* 0.001 for KPC-producing *K. pneumoniae* colonization during ICU stay).

Patient characteristics	Nonobese (*n* = 87)	Obese (*n* = 38)	*P*
*Demographics*			
Age (years)	60.5 ± 19.5	59.2 ± 15.2	0.464
Male gender	41 (62.1%)	14 (46.7%)	0.185
*Chronic diseases (number)*	0.9 ± 0.9	1.1 ± 1.1	0.242
Diabetes mellitus	16 (18.2%)	11 (28.9%)	0.238
Chronic obstructive pulmonary disease	17 (19.5%)	11 (28.9%)	0.253
Chronic heart failure	12 (13.8%)	5 (13.2%)	1.000
Chronic renal failure requiring dialysis	8 (9.2%)	1 (2.6%)	0.274
Malignancy (solid organ or heamatologic one)	9 (10.3%)	6 (15.8%)	0.385
Cortisone use (within last month of ICU admission)	10 (11.5%)	6 (15.8%)	0.564
*Hospitalization data*			
Prior emergency surgery	18 (20.7%)	11 (28.9%)	0.360
Prior abdominal surgery	21 (24.1%)	13 (34.2%)	0.278
Prior hospitalization	59 (67.8%)	26 (68.4%)	1.000
APACHE II Score upon admission	18.7 ± 7.8	16.2 ± 7.8	0.169
SAPS II upon admission	42.8 ± 13.3	44.3 ± 13.2	0.658
SOFA score upon admission	9.0 ± 3.9	9.2 ± 3.8	0.786
ICU length of stay (days)	11.9 ± 12.6	24.9 ± 26.4	<0.001
Mortality	38 (43.7%)	29 (76.3%)	0.001^*∗*^
*ICU data*			
Cortisone	49 (56.3%)	31 (81.6%)	0.008
Antibiotics administered (number)	3.4 ± 1.5	4.9 ± 2.5	<0.001
Mean antibiotic use per day	2.8 ± 0.9	3.1 ± 0.9	0.005
Tracheostomy	31 (35.6%)	26 (68.4%)	0.001
Number of invasive catheters^a^	0.5 ± 0.8	1.9 ± 2.1	<0.001
Parenteral nutrition	19 (21.8%)	16 (42.1%)	0.030
Enteral nutrition	37 (42.5%)	25 (65.8%)	0.020
Dialysis	6 (6.9%)	10 (26.3%)	0.007
*Colonization/infection data*			
Site of infection			
Meningitis	9 (10.3%)	1 (2.6%)	0.280
Pneumonia	44 (50.6%)	18 (47.4%)	0.846
Intra-abdominal infection	15 (17.2%)	12 (31.6%)	0.098
Urinary-tract infection	13 (14.9%)	2 (5.3%)	0.148
Skin and soft tissue infection	6 (6.9%)	4 (10.5%)	0.490
Bloodstream infection (primary or secondary)	10 (11.5%)	12 (31.6%)	0.010^*∗*^
Septic shock	34 (39.1%)	21 (55.3%)	0.123
*Colonization data*			
KPC-Kp colonization during ICU stay	21 (24.1%)	23 (60.5%)	<0.001^*∗*^
VRE colonization during ICU stay	7 (8.0%)	4 (10.5%)	0.734

Data are number (%) of patients or mean ± SD.

ICU: intensive care unit; APACHE II: Acute Physiology and Chronic Health Evaluation II; SAPS: Simplified Acute Physiology Score II; SOFA: Sequential Organ Failure Assessment; KPC-Kp: KPC-producing *K. pneumoniae*; VRE: vancomycin-resistant *Enterococcus*.

^a^All patients after ICU admission were intubated and mechanically ventilated and were continuously monitored with a central venous catheter, an arterial catheter, and a urinary catheter. Number of catheters does not include the aforementioned catheters.

## Abbreviations

ICU: Intensive care unit
LOS: Length of stay.
